# Intraventricular immunovirotherapy; a translational step forward

**DOI:** 10.18632/oncotarget.28343

**Published:** 2023-01-12

**Authors:** Joshua D. Bernstock, Sarah Blitz, Kyung-Don Kang, Gregory K. Friedman

**Affiliations:** ^1^Department of Neurosurgery, Brigham and Women’s Hospital, Harvard Medical School, Boston, MA 02115, USA; ^2^David H. Koch Institute for Integrative Cancer Research, Massachusetts Institute of Technology, Cambridge, MA 02139, USA; ^3^Department of Pediatrics, Division of Pediatric Hematology and Oncology, University of Alabama at Birmingham, Birmingham, AL 35233, USA; ^*^These authors contributed equally to this work

**Keywords:** oncolytic virotherapy, herpes simplex virus (HSV), G207, intraventricular therapy, leptomeningeal disease

## Abstract

Oncolytic virotherapy with intratumoral engineered type-1 herpes simplex virus (HSV) has been proven safe with promising efficacy in recent clinical trials for treatment of both pediatric and adult high-grade glioma. However, this approach excludes patients with tumors in surgically inaccessible and/or eloquent brain regions. Current delivery methods are also unable to access/treat those patients with metastatic disease in the spinal cord and/or leptomeningeal disease. A recent preclinical study has paved the way for clinical translation of intraventricular administration of oHSV by identifying and mitigating the toxicity associated with this route for therapeutic benefit in murine models of disseminated medulloblastoma. This work may ultimately allow for targeting of intractable disease and provides a feasible option for the repetitive dosing of clinically relevant immunovirotherapy, G207.

## INTRODUCTION

Central nervous system (CNS) tumors, including primary tumors and metastatic or leptomeningeal disease (LMD), are associated with high morbidity and mortality in both adults and children [1–4]. Immunotherapies, including oncolytic virotherapy, offer novel, targeted approaches to treat brain tumors with increased efficacy and reduced toxicity [5]. Oncolytic herpes simplex virus type-1 (oHSV) has shown promise in clinical trials in both pediatric and adult brain tumors [6–9]. These completed trials all utilized intratumoral inoculation of virus via needles or catheters with convection-enhanced delivery or slow-rate infusion, allowing the virus to bypass the blood-brain barrier and facilitating maximum intratumoral concentrations with low systemic toxicity. However, direct inoculation requires an invasive neurosurgical procedure and may disqualify tumors in surgically inaccessible or eloquent brain regions as well as metastatic and/or LMD. Additionally, while repeated administration of virus has demonstrated encouraging effects on survival, it requires repeated neurosurgical procedures [6]. Direct injection into cerebral ventricles and/or intrathecal delivery has the potential to overcome the limitations of intratumoral delivery, but has since been avoided due to concerns of toxicity, even excluding patients where intratumoral administration would lead to ventricular breech [6–9].

**Figure 1:**
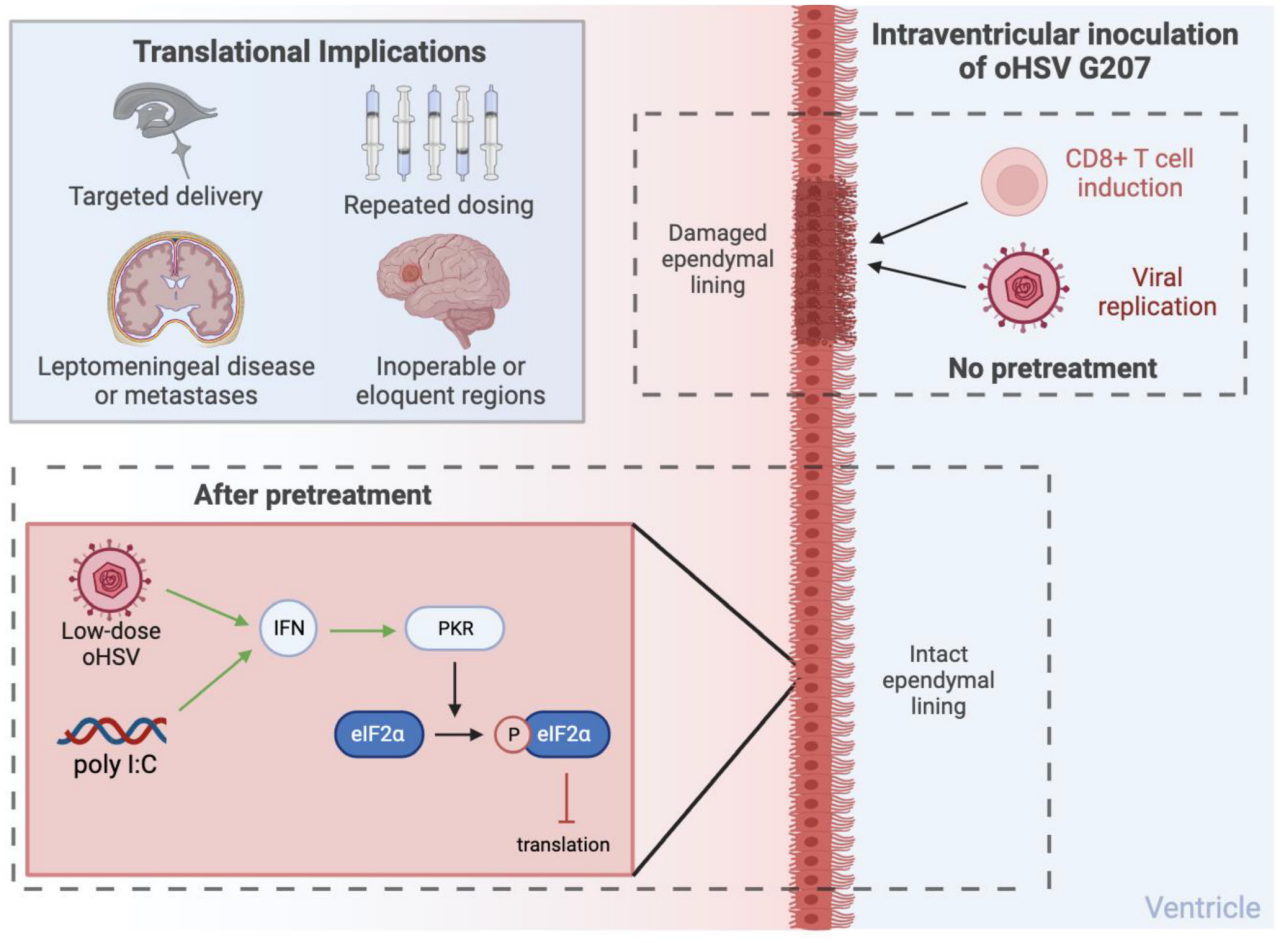
Putative mechanisms of toxicity. Damage of the ependymal lining can be mitigated via pretreatment with IVT low-dose oHSV or poly I:C. Translational implications of such an approach are also highlighted. Made with https://BioRender.com. Abbreviations: oHSV: oncolytic herpes simplex virus; poly I:C: polyinosinic-polycytidylic acid; IFN: interferon; PKR: protein kinase R; eIF2α: eukaryotic initiation factor-2α.

With the goal of maximizing the therapeutic potential of oHSV, Kang and Bernstock et al., inoculated ventricles of mice with *γ*_1_ *34.5*-deleted oHSV (G207) to identify mechanisms of damage with intraventricular therapy (IVT) and potential steps to mitigate toxicity [10]. They found that toxicity was due to damage to the ependymal lining, in part due to viral replication and induction of CD8^+^ T cells (Figure 1). They therefore hypothesized that the interferon (IFN)-induced protein kinase R phosphorylation of eukaryotic initiation factor-2α (eIF2α) is not initiated effectively in ependymal cells leading to toxicity [11]. As such the research team decided to employ a pretreatment paradigm with the potent IFN inducer polyinosinic-polycytidylic acid (poly I:C) or with IVT low-dose HSV in order to “prime” the ependymal cells. This approach with clinically-relevant oHSV G207 enabled safe delivery of multiple therapeutic doses and in so doing prolonged survival in human and murine metastatic medulloblastoma models.

The work has significant translational implications. G207 is the most extensively studied oHSV in preclinical and clinical brain tumor studies and has been proven safe in the CNS of both adults and children [6–9, 12–15]. In a completed phase I trial in children with recurrent/progressive supratentorial high-grade glioma, G207 was safe with promising efficacy results including prolonged responses in some patients and a median overall survival of 12.2 months, which compares favorably with historical survival of approximately 6 months for children at first relapse [9, 16]. This promising efficacy data will need to be confirmed in larger studies and has led to the development of a multi-institutional phase II trial (NCT04482933) in pediatric high-grade glioma at first relapse/progression, which is expected to open in early 2023. Additionally, there is an ongoing first-in-human phase I trial studying intratumoral G207 for recurrent malignant pediatric cerebellar brain tumors [17].

Critically, this preclinical study demonstrated the putative safety/efficacy of IVT G207 and in so doing highlighted a new route for inoculation; such work may allow for the targeting of previously unreachable diseases such as disseminated LMD or metastases as well as tumors in inoperable and/or eloquent regions. This has the potential to expand the variety of tumor types and locations targetable by G207 and increase opportunities for patients that traditionally have had very limited options. Addressing metastases may also lead to lower and more focused doses of radiation thereby reducing treatment-related toxicity, as radiation and oHSV appear to have a synergistic role [18, 19]. Accordingly, we feel that such studies support future clinical trials of G207 through this approach.

IVT would also pave the way for an accessible route for repeated doses of virus as compared to multiple invasive neurosurgical procedures. Increased dosing may increase the efficacy of virotherapy or extend the clinical benefit for longer time periods. A preclinical trial of G207 demonstrated superior efficacy of six intratumoral doses over one with a tenfold higher dose [20]. A recent phase II clinical trial of G47Δ in adults with residual/recurrent supratentorial glioblastoma used a maximum of six intratumoral doses, and patients had a median survival of 20.2 months after virotherapy initiation, which exceeds historical survival in this patient population [6]. Although intravenous delivery of oHSV was safe with some evidence of viral replication in extracranial solid tumors [21], hurdles such as neutralizing antibodies, the blood-brain barrier, and first-pass metabolism prevent adequate virus from reaching intracranial tumors to generate sufficient response. Preclinical trials have attempted to overcome these challenges through a variety of techniques, but have not yet been tested in patients [22–25]. Intraventricular or intrathecal inoculation bypasses many of these barriers allowing for more targeted administration.

Overall, demonstrating the safety and efficacy of IVT with G207 is a significant step towards expanding the capabilities of oHSV, paving the way for new clinical trials, and increasing the potential of an already promising therapy.
